# Report of Deaths in the City of Buffalo for the Month of April, 1864

**Published:** 1864-06

**Authors:** Sandford Eastman

**Affiliations:** Health Physician


*Report of Deaths in the City of Buffalo for the month of April*, 1864.

Abscess, 1; Accident, 3; do. by burn, 3; do. by drowning, 10; Apoplexy, 1; Bronchitis, 3; Cancer stomach, 2; Consumption, 16; Convulsions, 9; Croup, diphtheritic, 4; Croup, 2; Debility, 3; Dentition, 1; Diarrhœa, 1; Disease of the brain, 3; do. heart, 3; do. liver, 1; Diphtheria, 4; Dropsy, 2; Effects of frost bite, 1; Empyema, 1; Erysipelas, 2; Fever, 1; do. puerperal, 4; do. puerperal hysteria, 1; do. scarlet, 5; do. typhoid, 10; Hæmorrhage from lungs, 1; Inflammation of Brain, 1; do. brain and meninges, 3; do. lungs, 9; do. lungs typhoid, 4; do. veins of womb, 1; Intemperance, 3; Intussusception, 1; Marasmus, 4; Ovarian Tumor, 1; Old age, 2; Paralysis, 1; Pyæmia, 2; Scrofula, 1; Suicide, (poison,) 1; Tabes Mesenterica, 2; Ulceration in Bowels, 1; Unknown, 3; Uræmia, 1; Still-born, 5. Males 77. Females, 67.

*Locality.*—City at large, 128; Hospital of Sisters of Charity, 8; Buffalo General Hospital, 1; Catholic Foundling Asylum, 3; Protestant Orphan Asylum, 1; Erie County Alms House, 3.

*By whom Certified.*—By Regular Physicians at Public Institutions, 16; by Regular Physicians in city at large, 74; by Irregular Practitioners, 16; by Coroner, 18; by Undertakers, 20. Total, 144.

SANDFORD EASTMAN, M. D., Health Physician.

